# Effectiveness of solid and liquid biogas-derived organic fertilizers on growth, yield, and nutrient status of arugula in calcareous soils

**DOI:** 10.7717/peerj.21707

**Published:** 2026-09-14

**Authors:** Ahmet Safak Maltas

**Affiliations:** Finike Vocational School, Department of Plant and Animal Production, Akdeniz University, Antalya, Turkey

**Keywords:** Cattle manure, Poultry manure, Solid-liquid manure, *Eruca sativa*

## Abstract

This study aimed to evaluate the effectiveness of solid and liquid organic fertilizers derived from biogas waste in crop production, with the goal of promoting their use as sustainable fertilizers. The experimental design included a control, two doses of liquid organic fertilizer (200 L ha^−1^ and 400 L ha^−1^), and three doses of solid organic fertilizer (0, 3,000 kg ha^−1^, and 6,000 kg ha^−1^). The combined application of solid and liquid organic fertilizers promoted faster plant height growth in arugula (*Eruca sativa*) within the same growth period, thereby enabling earlier harvest. Solid and liquid organic fertilizer applications had a significant effect on plant tissue concentrations of nitrogen, phosphorus, potassium, calcium, magnesium, iron, zinc, manganese, and copper. Significant period and treatment means were detected for phosphorus, magnesium, zinc, and copper, while potassium, calcium, and magnesium were significantly influenced by period alone. The highest manganese and iron concentrations were achieved exclusively with the liquid organic fertilizer treatments. Soil analyses revealed that the treatments significantly affected total nitrogen, available phosphorus, exchangeable magnesium, available iron, zinc, manganese, and copper levels, as well as soil pH, electrical conductivity (EC), and organic matter content. The results indicate that, in short-cycle crops such as arugula, liquid organic fertilizers applied through fertigation are generally more effective than solid forms. The use of low-compaction pelletized or powdered formulations is recommended to enhance the effectiveness of solid organic fertilizers.

## Introduction

In today’s fast-paced and convenience-oriented lifestyle, the demand for salad crops such as *Eruca sativa*, which can be consumed fresh without processing, is steadily increasing. In particular, consumer interest in organically produced vegetables has grown markedly in recent years, in parallel with the rising emphasis on healthy diets. However, arugula are among the leafy vegetables that accumulate the highest nitrate levels, posing a potential risk to consumer health (Santamaria, 2006; Kyriacou et al., 2019; Guffanti et al., 2022). Due to their slow-release characteristics, organic fertilizers do not lead to an increase in the nitrate content of plant leaves (Ceylan et al., 2017).

Organic fertilizers are a key input influencing yield and quality in crop production. Nevertheless, the direct use of these fertilizers in agriculture, without prior processing, is associated with several drawbacks, including the introduction of weed seeds, dissemination of pathogenic fungi and bacteria, and variability in their chemical composition (Pleasant & Schlather, 1994; Abawi & Widmer, 2000; Manogaran et al., 2022). Therefore, processing organic fertilizers to eliminate harmful properties and to standardize their composition (*e.g.*, nutrient content, pH, electrical conductivity) is a more effective and safer approach for agricultural applications (Lazarovits, Conn & Potter, 1999; McKellar & Nelson, 2003; Thepsilvisut et al., 2022; Maltaş, 2025a; Maltaş, 2025b).

Recycling is an essential component of sustainable living. Currently, organic waste is recycled through various methods, and new approaches are being explored for their utilization in different sectors. Organic wastes can be converted into biogas energy under anaerobic conditions through the activity of diverse groups of microorganisms (Mao et al., 2015). Following energy extraction during biogas production, the residual organic matter can be utilized as an organic fertilizer in agriculture—a practice that is of particular significance for resource efficiency and sustainability. In this context, evaluating the agricultural potential of organic fertilizers derived from post-energy residues of biogas plants is of considerable importance.

There are numerous studies examining the use of biogas plant–derived organic fertilizers in agriculture (Alengebawy et al., 2024; Alhammad & Seleiman, 2023). However, research addressing the combined use of solid and liquid fractions derived from biogas plants remains limited. Solid organic fertilizers are generally associated with longer-term effects through gradual nutrient release and improvements in soil organic matter, whereas liquid organic fertilizers applied through drip irrigation can provide nutrients more rapidly to plants.

Despite these complementary characteristics, information on the integrated application of both biogas fertilizer fractions, particularly in short-cycle vegetable crops, is still scarce. Short-cycle crops require rapid nutrient availability to sustain fast growth, and therefore the simultaneous use of both fractions may provide synergistic benefits in terms of nutrient supply dynamics and soil fertility enhancement. The solid fraction may contribute to soil organic matter accumulation and sustained nutrient release, while the liquid fraction can supply readily available nutrients for immediate plant uptake.

Accordingly, evaluating the integrated use of both biogas fertilizer fractions may provide a more comprehensive understanding of their agronomic potential. In this context, the present study investigates the combined effects of solid and liquid biogas fertilizers on growth and yield parameters of a short-cycle crop under greenhouse conditions, thereby contributing new insights into sustainable nutrient management strategies.

## Materials and Methods

This study was conducted over two consecutive cropping periods as a fixed-plot experiment in the “No. 1 Glass Greenhouse” of the Akdeniz University Faculty of Agriculture Farm Directorate (36°53′54.50″N 30°38′19″E). The experiment was arranged in a randomized complete block design with seven treatments and three replications, for a total of 21 plots. Solid and liquid organic fertilizers derived from a biogas production system using a mixture of cattle manure (60%) and poultry manure (40%) were used as nutrient sources in the experiment. The nutrient compositions of the solid and liquid organic fertilizers applied in the study are provided in Table 1. Application rates for the liquid and solid organic fertilizers were determined based on agronomic conditions, crop nutrient requirements, and the economic considerations of arugula production (Table 2). The soil used in the experiment and its selected physical and chemical properties are presented in Table 3.

**Table 1:** Some chemical properties of liquid and solid manures used in the experiment, as reported by the companies.

Parameter	Manure type	
	Liquid	Solid
Organic matter (%)	20	50
Total N (%)	3	2
Total P (%)	0.7	2
Total K (%)	5	1
Dry matter (%)	40	80
pH	6–7	5.5–7.5

**Table 2:** Treatments in the experiment.

Treatment	
A0	Control
A1	0 kg ha^−1^ SM and 200 L ha^−1^ LM
A2	3,000 kg ha^−1^ SM and 400 L ha^−1^ LM
A3	6,000 kg ha^−1^ SM and 200 L ha^−1^ LM
A4	0 kg ha^−1^ SM and 400 L ha^−1^ LM
A5	3,000 kg ha^−1^ SM and 200 L ha^−1^ LM
A6	6,000 kg ha^−1^ SM and 400 L ha^−1^ LM

**Notes.**

A Application SM Solid manure LM Liquid manure

**Table 3:** Some physical and chemical properties of the experimental soil.

Parameter	Content	Parameter	Content
Texture	Clay loam	Exchangeable potassium (K_2_O kg ha^−1^)	936.3
pH	7.54	Exchangeable calcium (CaO kg ha^−1^)	8,203.2
EC (dS m^−1^)	0.70	Exchangeable magnesium (MgO kg da^−1^)	898.8
Lime (%)	17.4	Available iron (ppm)	3.8
Organic matter (%)	2.7	Available manganese (ppm)	10.6
Total N (%)	0.14	Available zinc (ppm)	9.2
Available phosphorus (P_2_O_5_ kg ha^−1^)	301.7	Available cupper (ppm)	6.9

Following primary tillage, raised beds were formed using a tractor and subsequently leveled manually with shovels. Each plot measured 1.20 m in width and 3.20 m in length, with an inter-plot spacing of 0.80 m. Immediately after the plots were prepared, solid fertilizer treatments were applied, and the soil was left for a 10-day incubation period. Arugula plants (Bengi F1) were established from direct seed sowing. The study was conducted in fixed experimental plots during two periods. After the first growing period, solid organic fertilizers were reapplied to the plots, followed by soil tillage and a 10-day incubation period. In the second growing period, arugula seeds were sown again. In both periods, harvesting was performed 45 days after sowing. Irrigation was applied simultaneously and at equal amounts to all plots in each growing period. The average greenhouse temperature and relative humidity during the first and second growing seasons were 18.23 °C and 61.45%, and 15.87 °C and 67.12%, respectively.

In both growing periods, liquid organic fertilizer was applied weekly, beginning after seed germination and continuing until harvest. A total of five applications were performed during each growing period. Accordingly, the seasonal application rates of 200 and 400 L ha^−^^1^ were evenly distributed across the five weekly applications, corresponding to 40 and 80 L ha^−^^1^ per application, respectively. Furthermore, no chemical pesticides or synthetic fertilizers were used to ensure compliance with organic production standards.

**Figure 1:**
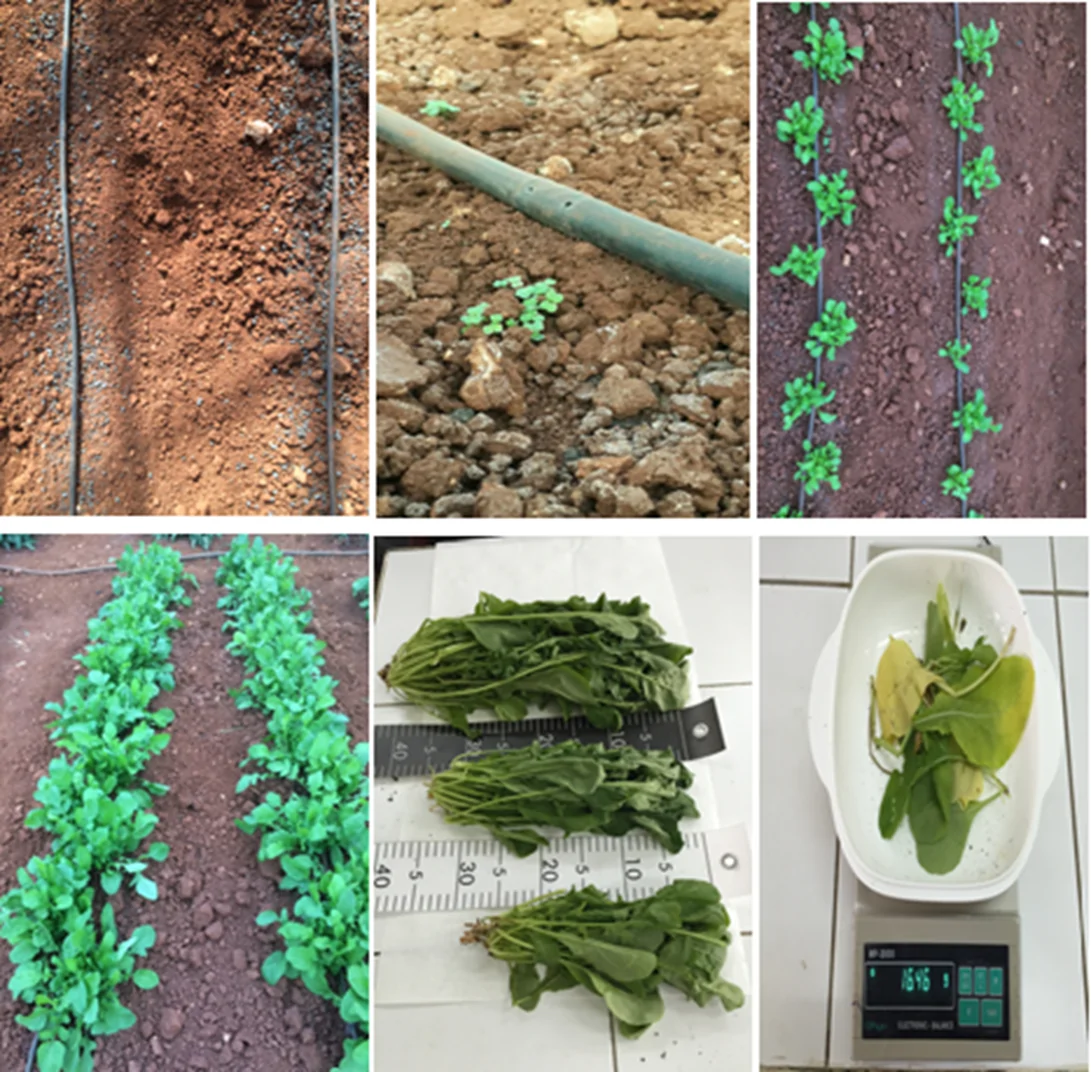
Experimental setup and major stages of the study, including field preparation, application of biogas-derived organic fertilizers, plant growth, and measurement procedures.

For pest management, insect-proof nets were installed on the greenhouse ventilation openings. For disease management, an internal circulation fan was operated during humid weather conditions to improve air movement. Photographs documenting various stages of the experiment are presented in Fig. 1.

### Yield and quality parameters

The roots and shoots of the arugula plants were separated, and the samples were transferred to the Laboratory of Soil Science and Plant Nutrition, Faculty of Agriculture, Akdeniz University. All measured values were converted to a per-hectare basis according to plot size and recorded as yield and quality parameters.

For the determination of yellow leaf number and weight, leaves that were unmarketable in color (particularly yellow leaves) were selected, counted, and weighed (Fig. 1). For marketable yield, green leaves were counted and weighed. After recording the marketable yield, plant height was measured (Fig. 1). The samples were then oven-dried at 65 °C for 48 h until a constant weight was achieved, and dry weight was recorded. The dried plant material was ground, and nutrient element analyses were performed on these samples.

### Soil analysis

Soil samples were collected at the time of harvesting from a depth of 0 to 20 cm. Soil physical and chemical parameters that were measured included organic matter content (Black, 1965), EC and pH (1/2.5 = soil water ratio) (Jackson, 1967), lime content (Caglar, 1949), texture (Bouyoucos, 1951), total nitrogen (Kacar, 1995), available phosphorus (Olsen & Sommers, 1982) and exchangeable calcium (Ca), potassium (K) and magnesium (Mg) (Kacar, 1995). Available iron (Fe), zinc (Zn), manganese (Mn), and copper (Cu) contents were determined with the DTPA method (Lindsay & Norvell, 1978; Perkin Elmer ICP-OES 7100DV).

### Plant analysis

The total nitrogen content of arugula plants was determined using the modified Kjeldahl method, while phosphorus, potassium, calcium, magnesium, iron, zinc, manganese, and copper contents were determined by wet digestion. Analyses of macro- and micronutrients were carried out according to the procedures described by Kacar & Inal (2008), and measurements were performed using a Perkin Elmer ICP-OES 7100DV instrument.

### Statistical analysis

The experiment was arranged in a randomized complete block design (RCBD) with three replications, each comprising 15 arugula plants, and the data were analyzed accordingly. All statistical analyses were performed through XLSTAT (version 2016.02.28451, Addinsoft, France). The Least Significant Difference (LSD) test was utilized for the comparison of means (*p* ≤ 0.05).

## Results

### Macronutrient content in plants

The effects of solid and liquid organic fertilizer applications on the macronutrient composition of arugula are summarized in Table 4. Treatment means significantly influenced plant tissue nitrogen, phosphorus, potassium, calcium, and magnesium concentrations. In addition, growing period effects were significant for potassium, calcium, and magnesium levels. Notably, both solid and liquid organic fertilizer applications resulted in a consistent reduction in plant tissue magnesium content across both growing periods.

### Micronutrient content in plants

The effects of solid and liquid organic fertilizer applications on the micronutrient composition of arugula are presented in Table 5. Treatment means had statistically significant effects on iron, zinc, manganese, and copper concentrations.

**Table 4:** Effects of treatments on macronutrient content of arugula plants.

Application	N (%)			P (%)			K (%)			Ca (%)			Mg (%)		
	1st	2nd	Mean	1st	2nd	Mean	1st	2nd	Mean	1st	2nd	Mean	1st	2nd	Mean
A0	6.11 ± 0.14	5.81 ± 0.18	5.96 ± 0.12cd	0.35 ± 0.02bcd	0.32 ± 0.02d	0.33 ± 0.01c	2.34 ± 0.00	1.92 ± 0.05	2.13 ± 0.07d	1.89 ± 0.15	2.19 ± 0.09	2.04 ± 0.11ab	0.18 ± 0.00a	0.20 ± 0.01a	0.19 ± 0.01a
A1	5.92 ± 0.01	6.01 ± 0.04	5.97 ± 0.03cd	0.35 ± 0.01bcd	0.31 ± 0.02d	0.33 ± 0.01c	2.42 ± 0.07	2.31 ± 0.06	2.36 ± 0.05c	1.75 ± 0.16	2.21 ± 0.04	1.98 ± 0.18bc	0.13 ± 0.00b	0.16 ± 0.01bc	0.15 ± 0.01b
A2	6.07 ± 0.04	6.17 ± 0.09	6.12 ± 0.05bc	0.38 ± 0.01ab	0.32 ± 0.01d	0.35 ± 0.01ab	2.52 ± 0.01	2.43 ± 0.05	2.48 ± 0.03c	2.02 ± 0.38	2.41 ± 0.06	2.22 ± 0.19ab	0.13 ± 0.01b	0.15 ± 0.01c	0.14 ± 0.01b
A3	6.56 ± 0.00	6.59 ± 0.14	6.57 ± 0.07a	0.37 ± 0.01abc	0.34 ± 0.01cd	0.36 ± 0.01ab	2.99 ± 0.04	2.92 ± 0.07	2.96 ± 0.04b	2.02 ± 0.31	2.26 ± 0.13	2.14 ± 0.16ab	0.14 ± 0.00b	0.15 ± 0.01c	0.15 ± 0.01b
A4	5.63 ± 0.06	5.92 ± 0.24	5.78 ± 0.01d	0.26 ± 0.00e	0.32 ± 0.02d	0.29 ± 0.02d	3.21 ± 0.13	3.16 ± 0.06	3.18 ± 0.07a	2.28 ± 0.04	2.43 ± 0.18	2.36 ± 0.09a	0.17 ± 0.00a	0.18 ± 0.01ab	0.18 ± 0.00a
A5	6.15 ± 0.01	5.99 ± 0.12	6.07 ± 0.06bcd	0.35 ± 0.01bcd	0.37 ± 0.01abc	0.36 ± 0.01ab	3.09 ± 0.09	3.23 ± 0.06	3.16 ± 0.06a	1.18 ± 0.04	2.09 ± 0.13	1.64 ± 0.21c	0.13 ± 0.00b	0.14 ± 0.01c	0.14 ± 0.01b
A6	6.20 ± 0.03	6.49 ± 0.11	6.35 ± 0.08ab	0.34 ± 0.02bcd	0.40 ± 0.01a	0.37 ± 0.02a	2.93 ± 0.04	2.91 ± 0.02	2.92 ± 0.02b	1.31 ± 0.017	1.96 ± 0.04	1.64 ± 0.17c	0.13 ± 0.00b	0.14 ± 0.00c	0.14 ± 0.00b
Significance (treatment)	ns	ns	^**^	^*^	^**^	^**^	ns	ns	^**^	ns	ns	^*^	^*^	^*^	^**^
Minimum	5.63	5.81	5.72	0.26	0.31	0.29	2.23	1.92	2.13	1.18	1.96	1.57	0.13	0.14	0.14
Maximum	6.56	6.59	6.58	0.38	0.40	0.39	3.21	3.23	3.22	2.28	2.41	2.35	0.18	0.20	0.19
Mean	6.09 ± 0.06	6.14 ± 0.08	6.11 ± 0.06	0.34 ± 0.01	0.34 ± 0.01	0.34 ± 0.01	2.79 ± 0.08a	2.70 ± 0.10b	2.74 ± 0.06	1.78 ± 0.11b	2.22 ± 0.05a	2.00 ± 0.08	0.14 ± 0.01b	0.16 ± 0.01a	0.15 ± 0.01
Significance (period)	ns			ns			^**^			^**^			^***^		

**Notes.**

Values followed by different lowercase letters within the same column are significantly different according to the LSD test at *p* ≤ 0.05.

* Significant at *P* ≤ 0.05.

** Significant at *P* ≤ 0.01.

*** Significant at *P* ≤ 0.001.

ns not significant

**Table 5:** Effects of treatments on micronutrient content of arugula plants.

Application	Fe (mg kg^−1^)			Zn (mg kg^−1^)			Mn (mg kg^−1^)				Cu (mg kg^−1^)		
	1st	2nd	Mean	1st	2nd	Mean	1st	2nd	Mean		1st	2nd	Mean
A0	75.06 ± 3.35	84.19 ± 3.54	79.62 ± 2.99c	15.31 ± 0.02b	16.19 ± 0.64ab	15.75 ± 0.35a	17.57 ± 0.26	18.48 ± 0.80	18.02 ± 0.43d	2.46 ± 0.19bc		1.88 ± 0.18de	2.17 ± 0.18cd
A1	91.47 ± 1.48	93.84 ± 2.33	92.65 ± 1.34c	12.76 ± 0.46c	12.23 ± 0.33c	12.49 ± 0.28c	17.79 ± 0.52	17.53 ± 0.66	17.66 ± 0.38d	2.28 ± 0.14bcd		2.40 ± 0.14bc	2.34 ± 0.09bc
A2	77.74 ± 16.23	84.80 ± 10.82	81.27 ± 8.87c	16.37 ± 0.45ab	15.26 ± 0.22b	15.81 ± 0.33a	19.22 ± 0.88	19.95 ± 0.14	19.58 ± 0.43c	2.69 ± 0.00ab		2.43 ± 0.02bc	2.56 ± 0.06ab
A3	110.48 ± 6.34	112.10 ± 7.87	111.29 ± 4.53b	13.54 ± 0.29c	13.43 ± 0.66c	13.48 ± 0.32bc	18.47 ± 0.10	16.40 ± 0.04	17.44 ± 0.47d	1.81 ± 0.15e		1.86 ± 0.06de	1.83 ± 0.07e
A4	145.60 ± 5.63	132.82 ± 2.44	139.21 ± 3.96a	13.43 ± 0.42c	12.72 ± 0.96c	13.08 ± 0.47c	22.97 ± 0.53	23.46 ± 0.64	23.21 ± 0.39a	1.79 ± 0.19e		2.08 ± 0.18cde	1.93 ± 0.13de
A5	77.61 ± 8.71	94.28 ± 5.03	85.95 ± 4.82c	13.31 ± 0.22c	15.64 ± 0.44b	14.48 ± 0.57b	19.44 ± 0.67	20.97 ± 0.45	20.21 ± 0.40c	2.06 ± 0.02de		2.02 ± 0.23cde	2.04 ± 0.11de
A6	97.46 ± 5.51	93.75 ± 5.97	95.61 ± 3.73c	15.43 ± 0.57b	17.41 ± 0.62a	16.42 ± 0.58a	21.47 ± 0.30	22.11 ± 0.17	21.79 ± 0.21b	2.21 ± 0.03cde		3.07 ± 0.06a	2.64 ± 0.20a
Significance (treatments)	ns	ns	^*^	^**^	^**^	^**^	ns	ns	^*^	^**^		^**^	^**^
Minimum	75.06	84.80	79.93	12.76	12.23	12.50	17.57	16.40	16.99	1.79		1.86	1.83
Maximum	145.60	132.82	139.21	16.37	17.41	16.89	22.97	23.46	23.22	2.69		3.07	2.88
Mean	96.50 ± 5.63	99.40 ± 4.09	97.95 ± .4.96	14.31 ± 0.34	14.70 ± 0.44	14.51 ± 0.36	19.56 ± 0.45	19.84 ± 0.55	19.70 ± 0.46	2.19 ± 0.08		2.25 ± 0.10	2.22 ± 0.07
Significance (period)	ns			ns			ns			ns			

**Notes.**

Values followed by different lowercase letters within the same column are significantly different according to the LSD test at *p* ≤ 0.05.

* Significant at *P* ≤ 0.05.

** Significant at *P* ≤ 0.01.

ns not significant

Although organic fertilizer treatments increased the fresh weight of arugula, no corresponding reduction in plant tissue micronutrient concentrations was observed. For manganese and iron, the highest concentrations were recorded in the A4 treatment.

### Soil macronutrient content

The effects of solid and liquid organic fertilizer applications on soil macronutrient composition are presented in Table 6. Treatment means had statistically significant effects on total nitrogen, available phosphorus, and exchangeable magnesium levels in the soil.

### Soil pH, EC, organic matter, and micronutrient content

The effects of solid and liquid organic fertilizer applications on soil chemical properties and micronutrient composition are presented in Table 7. Treatment means had statistically significant effects on soil pH, electrical conductivity (EC), organic matter content, and the concentrations of available iron, zinc, manganese, and copper.

**Table 6:** Effects of treatments on macronutrient content of soil.

Application	N (%)			P (mg kg^−1^)			K (meq 100 g^−1^)			Ca (meq 100 g^−1^)			Mg (meq 100 g^−1^)		
	1st	2nd	Mean	1st	2nd	Mean	1st	2nd	Mean	1st	2nd	Mean	1st	2nd	Mean
A0	0.11 ± 0.01	0.10 ± 0.01	0.11 ± 0.01c	196.34 ± 6.68	190.09 ± 5.80	193.22 ± 4.20d	1.45 ± 0.05	1.42 ± 0.07	1.44 ± 0.03	21.76 ± 0.07	21.77 ± 0.28	21.77 ± 0.13	3.78 ± 0.08	3.72 ± 0.05	3.75 ± 0.04abc
A1	0.11 ± 0.02	0.11 ± 0.01	0.11 ± 0.01c	191.92 ± 9.19	188.63 ± 11.49	190.27 ± 6.62d	1.39 ± 0.02	1.37 ± 0.04	1.38 ± 0.03	21.86 ± 0.22	21.76 ± 0.42	21.81 ± 0.21	3.67 ± 0.04	3.62 ± 0.10	3.65 ± 0.04bc
A2	0.20 ± 0.02	0.19 ± 0.03	0.20 ± 0.02a	214.82 ± 12.26	217.38 ± 11.42	216.10 ± 7.52bc	1.36 ± 0.02	1.34 ± 0.04	1.35 ± 0.02	21.60 ± 0.60	21.17 ± 0.56	21.39 ± 0.38	3.77 ± 0.08	3.78 ± 0.10	3.78 ± 0.05abc
A3	0.21 ± 0.01	0.22 ± 0.02	0.21 ± 0.01a	228.14 ± 2.22	225.94 ± 3.94	227.04 ± 2.08ab	1.36 ± 0.03	1.41 ± 0.07	1.39 ± 0.03	21.67 ± 1.05	21.75 ± 0.93	21.71 ± 0.63	3.72 ± 0.10	3.66 ± 0.12	3.69 ± 0.05bc
A4	0.13 ± 0.01	0.12 ± 0.02	0.13 ± 0.01c	231.33 ± 4.30	204.80 ± 5.66	209.07 ± 3.71c	1.38 ± 0.04	1.36 ± 0.07	1.37 ± 0.03	21.33 ± 1.18	21.50 ± 1.32	21.42 ± 0.78	3.57 ± 0.10	3.55 ± 0.17	3.56 ± 0.08c
A5	0.18 ± 0.01	0.16 ± 0.01	0.17 ± 0.01b	223.04 ± 4.86	216.47 ± 10.56	219.75 ± 5.40bc	1.38 ± 0.04	1.37 ± 0.06	1.38 ± 0.03	21.58 ± 0.75	21.65 ± 0.72	21.62 ± 0.46	3.82 ± 0.11	3.76 ± 0.15	3.79 ± 0.07ab
A6	0.20 ± 0.01	0.21 ± 0.02	0.20 ± 0.01a	238.16 ± 4.44	239.72 ± 6.09	238.94 ± 3.39a	1.40 ± 0.02	1.49 ± 0.05	1.45 ± 0.03	21.42 ± 0.99	21.69 ± 0.92	21.56 ± 0.62	3.95 ± 0.07	3.92 ± 0.13	3.94 ± 0.07a
Significance (treatment)	ns	ns	^**^	ns	ns	^*^	ns	ns	ns	ns	ns	ns	ns	ns	^**^
Minimum	0.11	0.10	0.11	191.92	188.63	190.28	1.36	1.37	1.37	21.33	21.17	21.39	3.57	3.55	3.56
Maximum	0.21	0.22	0.22	238.16	239.72	238.94	1.45	1.49	1.45	21.86	21.77	21.81	3.95	3.92	3.94
Mean	0.16 ± 0.01	0.16 ± 0.01	0.16 ± 0.01	215.11 ± 4.10	211.86 ± 4.69	213.49 ± 4.42	1.39 ± 0.02	1.39 ± 0.02	1.39 ± 0.02	21.60 ± 0.26	21.64 ± 0.25	21.62 ± 0.25	3.76 ± 0.04	3.71 ± 0.04	3.74 ± 0.04
Significance (period)	ns			ns			ns			ns			ns		

**Notes.**

Values followed by different lowercase letters within the same column are significantly different according to the LSD test at *p* ≤ 0.05.

* Significant at *P* ≤ 0.05.

** Significant at *P* ≤ 0.01.

ns not significant

**Table 7:** Effects of treatments on soil pH, soil EC, soil organic matter and soil micronutrient content.

Application	pH			EC			Organic matter (%)			Fe (mg kg^−1^)			Zn (mg kg^−1^)			Mn (mg kg^−1^)			Cu (mg kg^−1^)		
	1st	2nd	Mean	1st	2nd	Mean	1st	2nd	Mean	1st	2nd	Mean	1st	2nd	Mean	1st	2nd	Mean	1st	2nd	Mean
A0	7.33 ±0.03	7.31 ±0.01	7.32 ±0.02cd	0.60 ±0.03	0.57 ±0.02	0.58 ±0.02ab	2.58 ±0.06	2.51 ±0.06	2.54 ±0.04c	2.80 ±0.29	2.97 ±0.41	2.89 ±0.23b	9.90 ±0.10	10.30 ±0.37	10.10 ±0.19c	10.80 ±0.13	10.32 ±1.55	10.56 ±0.70c	6.87 ±0.35	7.72 ±0.51	7.30 ±0.33c
A1	7.35 ±0.03	7.37 ±0.03	7.36 ±0.02c	0.58 ±0.04	0.55 ±0.02	0.57 ±0.02ab	2.51 ±0.05	2.52 ±0.02	2.51 ±0.02c	2.93 ±0.30	2.94 ±0.34	2.94 ±0.21b	9.93 ±0.27	10.31 ±0.38	10.12 ±0.22c	12.62 ±1.01	11.72 ±1.86	12.17 ±0.97bc	8.04 ±0.67	7.65 ±0.92	7.85 ±0.52bc
A2	7.45 ±0.03	7.42 ±0.07	7.43 ±0.04ab	0.58 ±0.04	0.61 ±0.02	0.60 ±0.02a	2.55 ±0.03	2.55 ±0.03	2.55 ±0.02bc	3.00 ±0.33	3.13 ±0.60	3.07 ±0.31b	10.56 ±0.05	10.40 ±0.65	10.49 ±0.30bc	15.29 ±0.56	15.09 ±1.53	15.19 ±0.73a	10.52 ±0.21	10.99 ±0.42	10.76 ±0.24a
A3	7.30 ±0.04	7.34 ±0.03	7.32 ±0.02cd	0.62 ±0.06	0.59 ±0.04	0.61 ±0.03a	2.63 ±0.06	2.69 ±0.03	2.66 ±0.03a	3.74 ±0.13	3.88 ±0.29	3.81 ±0.20a	12.32 ±0.29	11.10 ±0.12	11.71 ±0.31a	17.54 ±0.52	16.67 ±0.56	17.11 ±0.39a	10.35 ±0.62	10.61 ±0.85	10.48 ±0.47a
A4	7.48 ±0.02	7.46 ±0.01	7.47 ±0.02a	0.51 ±0.05	0.51 ±0.05	0.51 ±0.03b	2.49 ±0.06	2.47 ±0.03	2.48 ±0.03c	2.83 ±0.26	3.17 ±0.46	3.00 ±0.23b	10.42 ±0.06	9.95 ±0.08	10.19 ±0.11c	13.13 ±1.31	12.58 ±1.30	12.85 ±0.83b	8.63 ±0.26	8.41 ±0.61	8.52 ±0.30b
A5	7.34 ±0.02	7.39 ±0.02	7.37 ±0.02bc	0.60 ±0.05	0.64 ±0.02	0.62 ±0.03a	2.54 ±0.06	2.56 ±0.03	2.55 ±0.03bc	3.55 ±0.35	3.47 ±0.35	3.51 ±0.20ab	10.54 ±0.38	10.42 ±0.33	10.48 ±0.23bc	15.88 ±0.82	14.95 ±0.86	15.41 ±0.57a	9.57 ±0.33	10.18 ±0.57	9.88 ±0.32a
A6	7.26 ±0.02	7.29 ±0.02	7.28 ±0.01d	0.63 ±0.02	0.66 ±0.02	0.65 ±0.02a	2.62 ±0.02	2.66 ±0.02	2.64 ±0.02ab	3.99 ±0.27	4.16 ±0.49	4.08 ±0.27a	10.97 ±0.26	11.05 ±0.12	11.01 ±0.13b	16.95 ±0.86	17.73 ±0.50	17.34 ±0.48a	9.66 ±0.16	10.63 ±0.69	10.15 ±0.38a
Significance (treatment)	ns	ns	^**^	ns	ns	^**^	ns	ns	^**^	ns	ns	^**^	ns	ns	^**^	ns	ns	^**^	ns	ns	^**^
Minimum	7.26	7.29	7.28	0.51	0.51	0.51	2.49	2.47	2.88	2.80	2.94	2.89	9.90	9.95	10.10	10.80	10.32	10.56	6.87	7.65	7.30
Maximum	7.48	7.46	7.47	0.63	0.66	0.65	2.63	2.69	2.66	3.99	4.16	4.08	12.32	11.10	11.01	16.95	17.73	17.34	10.52	10.99	10.76
Mean	7.36 ±0.02	7.37 ±0.02	7.37 ±0.02	0.59 ±0.02	0.59 ±0.01	0.59 ±0.01	2.56 ±0.02	2.56 ±0.02	2.56 ±0.02	3.26 ±0.14	3.39 ±0.17	3.33 ±0.16	10.66 ±0.19	10.51 ±0.14	10.59 ±0.15	14.60 ±0.58	14.15 ±0.68	14.38 ±0.62	9.09 ±0.30	9.46 ±0.37	9.28 ±0.34
Significance (period)	ns			ns			ns			ns			ns			ns			ns		

**Notes.**

Values followed by different lowercase letters within the same column are significantly different according to the LSD test at *p* ≤ 0.05.

** Significant at *P* ≤ 0.01.

ns not significant

**Figure 2:**
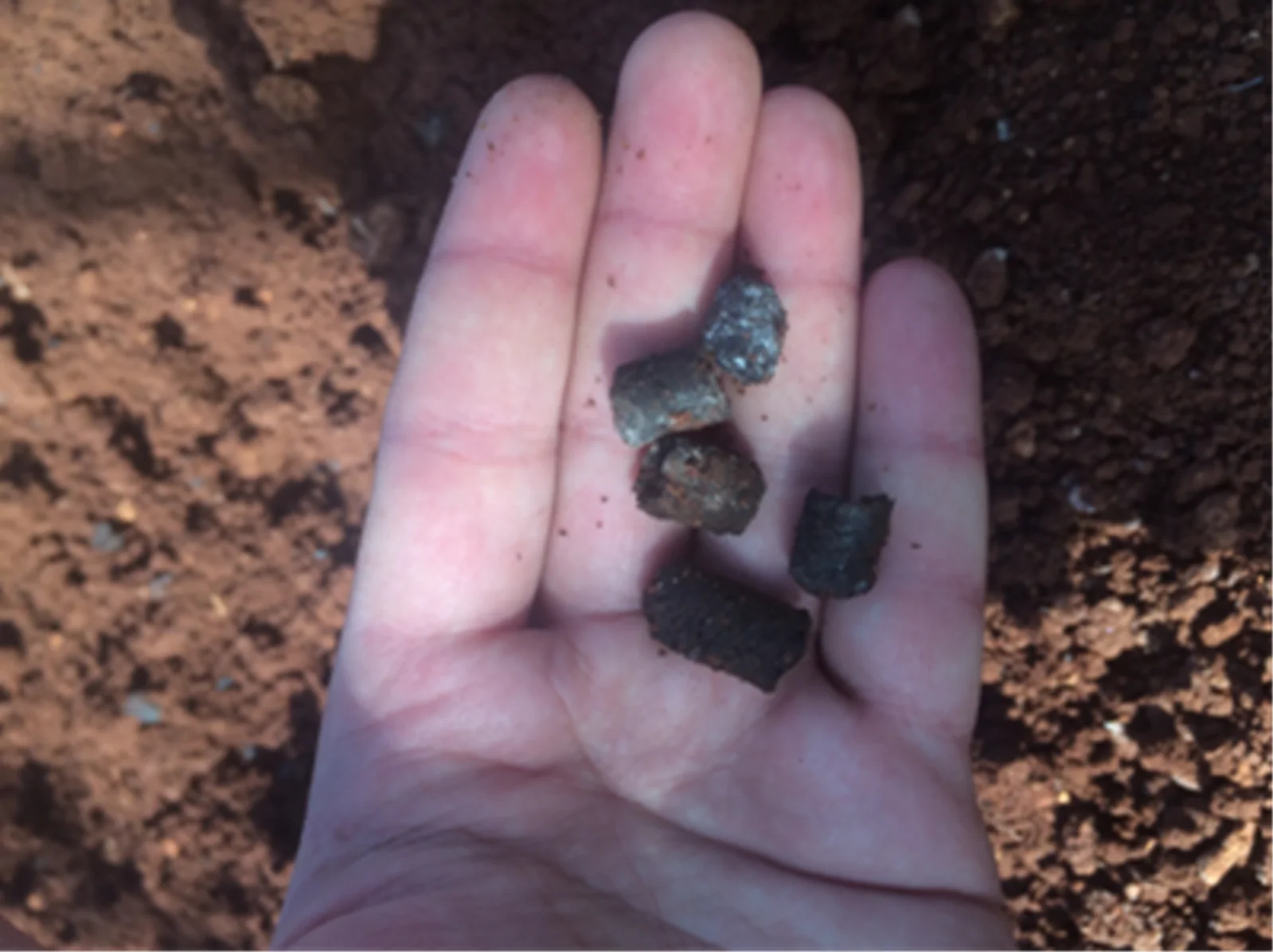
Residual solid organic fertilizer observed in the plots at the end of the growing season, indicating incomplete decomposition during the crop growth period.

In this study, soil salinity increased, particularly under solid organic fertilizer applications; however, this increase did not adversely affect arugula cultivation. On the contrary, solid organic fertilizers enhanced marketable yield and green leaf number in both cropping periods. At the end of both growing periods, a portion of the applied solid organic fertilizer remained visibly identifiable in the soil, indicating incomplete decomposition (Fig. 2).

Overall, organic fertilizer applications increased the availability of soil micronutrients, which was reflected in elevated micronutrient concentrations in arugula.

### Plant growth and yield

The effects of solid and liquid organic fertilizer applications on the harvest-related traits of arugula are presented in Table 8. Treatment means had statistically significant effects on marketable yield, dry weight, green leaf number, yellow leaf number, yellow leaf weight, and plant height. In addition, the growing period significantly influenced yellow leaf number.

Marketable yield increased by 62.25% to 118.59% compared to the control. All organic fertilizer applications also enhanced plant height, with increases ranging from 15.91% to 30.28% relative to the control.

## Discussion

This study demonstrates that biogas-derived organic fertilizers can effectively regulate soil nutrient dynamics and improve crop performance in calcareous soils. The results offer important evidence for the viability of integrated organic fertilization strategies as sustainable alternatives to conventional chemical inputs.

Co-processing poultry manure with cattle manure resulted in a solid organic fertilizer with an elevated phosphorus concentration. While the direct application of poultry manure may induce salinity-related constraints (Ata & Kaplan, 2020), its integration with cattle manure through biogas processing improved overall nutrient composition, particularly phosphorus. This enhancement translated into increased soil available phosphorus and higher phosphorus concentrations in arugula. Although phosphorus availability is typically restricted in calcareous soils due to fixation under high pH conditions (Wiedenfeld & SaulsJ, 2008; Abbaszadeh-Dahaji, Masalehi & Akhgar, 2020), the combined application of solid and liquid organic fertilizers in this study maintained adequate phosphorus nutrition even in the absence of chemical inputs. This finding underscores the potential of integrated organic fertilization strategies to sustain phosphorus availability in high-pH soils while reducing reliance on synthetic fertilizers.

**Table 8:** Effects of treatments on marketable yield, dry weight, green leaf number, yellow leaf number, yellow leaf weight, and plant height of arugula plants.

Application	Marketable yield (t/ha)			Dry weight (t/ha)			Green leaf number (number/ha x1000)			Green leaf number (number/ha x 1000)			Yellow leaf weight (t/ha)			Plant height (cm)		
	1st	2nd	Mean	1st	2nd	Mean	1st	2nd	Mean	1st	2nd	Mean	1st	2nd	Mean	1st	2nd	Mean
A0	10.69 ±1.07e	10.61 ±0.11e	10.65 ±0.70e	0.84 ±0.03e	0.82 ±0.01c	0.83 ±0.03e	290.37 ±17.06c	300.22 ±3.65c	295.30 ±10.31c	66.17 ±5.22ab	59.26 ±4.52b	62.72 ±3.45b	0.19 ±0.04d	0.16 ±0.01c	0.18 ±0.01d	33.67 ±0.67c	31.33 ±0.88b	32.50 ±0.72c
A1	17.62 ±0.46d	17.79 ±0.04d	17.71 ±0.28d	1.35 ±0.02cd	1.38 ±0.01b	1.37 ±0.04d	441.48 ±20.26ab	444.45 ±3.87ab	442.97 ±12.43ab	127.41 ±13.47a	115.56 ±4.24a	121.49 ±8.25a	0.36 ±0.01bc	0.33 ±0.01b	0.35 ±0.01bc	43.00 ±2.65a	41.67 ±2.85a	42.34 ±1.75a
A2	19.84 ±0.54c	19.46 ±0.06c	19.65 ±0.35c	1.54 ±0.02bc	1.51 ±0.01ab	1.53 ±0.03bc	417.78 ±15.79ab	435.56 ±1.85ab	426.67 ±8.25ab	69.14 ±11.51ab	58.27 ±3.47b	63.71 ±6.87b	0.27 ±0.02cd	0.25 ±0.01cb	0.26 ±0.01cd	39.33 ±2.78ab	38.00 ±2.65b	38.67 ±1.73ab
A3	19.83 ±0.02c	17.88 ±0.02c	18.86 ±0.01d	1.44 ±0.02cd	1.38 ±0.02b	1.41 ±0.04cd	524.45 ±44.58a	506.67 ±7.53a	515.56 ±15.24a	78.03 ±8.35ab	74.07 ±3.36b	76.05 ±7.34b	0.31 ±0.03c	0.28 ±0.01cb	0.30 ±0.01c	38.67 ±1.68b	36.67 ±0.88ab	37.67 ±0.96b
A4	17.00 ±0.63d	17.56 ±0.04d	17.28 ±0.35d	1.25 ±0.02d	1.29 ±0.01b	1.27 ±0.06d	364.44 ±21.79b	367.41 ±5.16b	365.79 ±15.11b	55.31 ±13.93b	49.38 ±3.96b	52.35 ±6.14b	0.18 ±0.02d	0.17 ±0.01c	0.18 ±0.01d	40.00 ±2.52ab	38.67 ±2.40b	39.34 ±1.59ab
A5	23.75 ±0.31a	22.81 ±1.15a	23.28 ±0.57a	1.80 ±0.02a	1.74 ±0.01a	1.77 ±0.05a	471.11 ±39.02ab	435.56 ±7.27ab	453.34 ±12.47ab	124.45 ±8.75a	104.69 ±8.01a	114.57 ±9.72a	0.55 ±0.02a	0.46 ±0.02a	0.51 ±0.07a	38.67 ±0.88ab	39.33 ±1.86b	39.00 ±0.95ab
A6	21.26 ±0.62b	21.35 ±0.06b	21.30 ±0.37b	1.59 ±0.01b	1.63 ±0.01a	1.61 ±0.02b	474.08 ±22.61ab	468.15 ±3.26ab	471.12 ±9.01ab	101.73 ±6.41b	114.57 ±9.24a	108.15 ±7.40a	0.44 ±0.06ab	0.47 ±0.01a	0.46 ±0.03ab	37.67 ±1.20b	38.33 ±1.85b	38.00 ±1.01b
Significance (treatment)	^*^	^*^	^*^	^**^	^**^	^***^	^*^	^*^	^*^	^**^	^**^	^**^	^***^	^***^	^**^	^**^	^**^	^**^
Minimum	10.69	10.61	10.65	0.84	0.82	0.83	290.37	300.22	295.30	55.31	49.38	52.35	0.18	0.16	0.18	33.67	31.33	32.50
Maximum	23.75	22.81	23.28	1.80	1.74	1.77	524.45	506.67	515.56	127.41	115.56	121.49	0.55	0.47	0.51	43.00	41.67	42.34
Mean	18.33 ±0.87	18.21 ±0.83	18.27 ±0.85	1.40 ±0.06	1.39 ±0.07	1.40 ±0.06	426.24 ±12.42	422.86 ±10.48	424.55 ±10.12	88.89 ±8.23a	82.26 ±6.73b	85.58 ±7.62	0.33 ±0.03	0.31 ±0.02	0.32 ±0.02	38.71 ±0.84	37.76 ±0.93	38.24 ±0.87
Significance (period)	ns			ns			ns			^**^			ns			ns		

**Notes.**

Values followed by different lowercase letters within the same column are significantly different according to the LSD test at *p* ≤ 0.05.

* Significant at *P* ≤ 0.05.

** Significant at *P* ≤ 0.01.

*** Significant at *P* ≤ 0.001.

ns not significant

In contrast, both solid and liquid organic fertilizer applications were associated with reduced magnesium concentrations in plants. Despite sufficient soil magnesium levels, the increased leaf number observed under organic fertilization likely induced a dilution effect, whereby biomass accumulation outpaced nutrient uptake, resulting in lower magnesium concentrations on a per-leaf basis. Such dilution effects are common in rapidly growing leafy vegetables. Moreover, the gradual nutrient release characteristic of organic fertilizers may not coincide with peak nutrient demand during intensive vegetative growth, potentially leading to transient limitations in the uptake of mobile nutrients such as magnesium. Similar patterns have been reported in previous studies (Gent, 2002; Maltaş, 2025a; Maltaş, 2025b; Abd-Elmoniem et al., 2025).

Micronutrient dynamics further highlight the effectiveness of organic fertilization under calcareous soil conditions. Despite the well-documented limitation of micronutrient availability in high-pH and lime-rich soils (He et al., 2021), organic fertilizer applications increased plant tissue micronutrient concentrations without inducing a dilution effect, even with increased biomass. Notably, the highest manganese and iron concentrations were observed under the A4 treatment, indicating that liquid organic fertilizers may be more effective than solid forms in enhancing micronutrient nutrition in short-cycle crops. This likely reflects the faster nutrient availability of liquid formulations and their more direct interaction with the rhizosphere.

The behavior of soil macronutrients aligns with these observations. In calcareous soils, nitrogen is prone to losses through volatilization and transformation processes, while phosphorus readily precipitates into insoluble forms (Taalab et al., 2019; Wahba, Fawkia & Zaghloul, 2019; Penn & Camberato, 2019; Barrow, Debnath & Sen, 2020). Although pH-lowering amendments are often used to improve nutrient availability, their effectiveness is limited by the high buffering capacity of such soils (Wiedenfeld, 2011; Zhang et al., 2016). In this context, organic fertilizers—particularly low-pH liquid formulations applied *via* fertigation—appear to offer a more effective approach by delivering nutrients directly to the rhizosphere, reducing nitrogen losses, and enhancing nutrient use efficiency (Umar et al., 2020). Additionally, regular application of liquid organic fertilizers may enable the cultivation of leafy vegetables such as arugula without promoting excessive nitrate accumulation in leaf tissues (Martínez-Alcántara et al., 2016).

Soil chemical properties also responded to organic fertilization. Although soil salinity increased following solid organic fertilizer application, this rise was not sufficient to adversely affect plant growth. Nevertheless, it highlights the importance of managing application rates and frequencies, particularly in long-term systems. A portion of the solid organic fertilizer remained visibly undecomposed at the end of both growing periods, indicating limited mineralization during the crop cycle. This observation may be related to pellet compaction, which has been reported to reduce decomposition and nutrient release. However, because the physical properties and compaction ratio of the fertilizer pellets used in this study were not measured, this explanation should be regarded as a potential mechanism rather than a confirmed cause. Future studies should evaluate pellet physical characteristics to clarify their effects on decomposition dynamics and nutrient availability in short-cycle crops such as arugula.

Furthermore, the increases in both soil and plant tissue micronutrient concentrations can be attributed not only to the intrinsic micronutrient content of organic fertilizers but also to the release of chelating organic acids during mineralization (Delgado, Quemada & Villalobos, 2016), which improve micronutrient availability under high-pH conditions. These findings suggest that liquid organic fertilizers may be particularly advantageous in environments where nutrient availability is constrained, including calcareous soils and cooler growing periods.

From an agronomic perspective, the substantial increases in yield and growth parameters observed in this study suggest that arugula can be successfully produced under the evaluated greenhouse and calcareous soil conditions using biogas-derived organic fertilizers without synthetic fertilizer inputs. Achieving high productivity under fully organic fertilization is particularly relevant for leafy vegetable production. Although previous studies have reported lower nitrate accumulation in organically fertilized leafy vegetables (Abd-Elrahman et al., 2022), which may contribute to improved product quality (Santamaria, 2006), these attributes were not evaluated in the present study and therefore require further investigation. The greater plant height and increased leaf number observed under organic fertilization further indicate accelerated growth, suggesting the potential for earlier harvest and more efficient production cycles. Collectively, these results highlight the agronomic and environmental benefits of biogas-derived organic fertilizers and support their broader evaluation in other leafy vegetable cropping systems.

## Conclusion

This study demonstrated that solid and liquid organic fertilizers derived from biogas residues can significantly improve the growth and yield of arugula. Across both growing periods, all organic fertilizer treatments resulted in higher marketable yields than the control, indicating that arugula can be successfully cultivated under the evaluated greenhouse and calcareous soil conditions using biogas-derived organic fertilizers without synthetic fertilizer inputs. However, the increase in yellow leaf number across treatments suggests that additional post-harvest labor may be required depending on the harvesting method used.

Liquid organic fertilizers in particular accelerated plant height growth within the same cultivation period, indicating a faster physiological response relative to solid fertilizers. Producers applying liquid organic fertilizers should therefore adjust their production schedules to avoid delayed harvests, which may negatively affect leaf quality or increase the risk of accumulating undesirable compounds in leafy vegetables.

Despite these considerations, strategic planning of application timing and harvest management can maximize the agronomic benefits of both solid and liquid organic fertilizers. Overall, the use of biogas-derived organic fertilizers supports sustainable arugula production by reducing the need for synthetic fertilizers, enhancing soil fertility, and enabling environmentally friendly crop management.

##  Supplemental Information

10.7717/peerj.21707/supp-1 Supplemental Information 1 Raw data

## Additional Information and Declarations

### Data Availability

The following information was supplied regarding data availability: The raw measurements are available in the Supplemental File.
